# Gyrate Atrophy of the Choroid and Retina Diagnosed in Adulthood With a Homozygous *OAT* Variant: A Case Report Highlighting the Need for Long‐Term Care

**DOI:** 10.1155/crop/8380205

**Published:** 2026-04-28

**Authors:** Hitomi Taniai, Kei Mizobuchi, Takaaki Hara, Takaaki Hayashi, Tadashi Nakano

**Affiliations:** ^1^ Department of Ophthalmology, The Jikei University School of Medicine, Tokyo, Japan, jikei.ac.jp; ^2^ Department of Ophthalmology, Shinkawabashi Hospital, Tokyo, Japan

## Abstract

**Purpose:**

The purpose of this study is to report a case of gyrate atrophy of the choroid and retina (GA) in a patient with a homozygous *OAT* variant who was unable to continue treatment due to loss to follow‐up.

**Case Presentation:**

A 21‐year‐old female patient (JU1902) from South Asia was referred to The Jikei University Hospital for clinical and genetic evaluation. She underwent comprehensive ophthalmic examinations and whole‐exome sequencing. Genetic analysis identified a homozygous variant in the *OAT* gene: a c.272G > A (p.Gly91Glu). Fundus photography showed sharply demarcated circular areas of chorioretinal atrophy in the peripheral retina. Fundus autofluorescence imaging revealed hypoautofluorescence corresponding to these atrophic areas. Optical coherence tomography showed multiple intraretinal cystic spaces with preservation of the outer retinal layers, including the ellipsoid zone. Goldmann perimetry revealed a markedly constricted visual field, with the I‐2 isopter restricted to less than 5° and the I‐4 isopter within 10°–20°. Full‐field electroretinography demonstrated norecordable responses under all stimulus conditions. Biochemical testing indicated markedly elevated serum and urinary ornithine concentrations. Following the genetic and clinical diagnosis, vitamin B6 supplementation was initiated at 100 mg/day. However, serum ornithine levels remained markedly elevated. Although an increased dosage of vitamin B6 (200 mg/day) and an arginine‐restricted diet were considered, the patient voluntarily discontinued follow‐up visits and was subsequently lost to follow‐up.

**Conclusion:**

This case highlights the importance of continued treatment and follow‐up in adult patients with GA. Forming a patient‐centered multidisciplinary team, including professional interpreters and cultural liaisons, is essential for ensuring patient understanding, treatment adherence, and long‐term visual preservation.

## 1. Introduction

Gyrate atrophy of the choroid and retina (GA) is an extremely rare inherited retinal dystrophy, with an estimated prevalence of approximately 1 in 1,500,000. It is classified as a congenital disorder of amino acid metabolism caused by pathogenic variants in the *ornithine aminotransferase* (*OAT*) gene. Ocular manifestations typically begin at birth and include night blindness, reduced visual acuity, and visual field constriction.

In 1988, the *OAT* gene, located on chromosome 10q26 and consisting of 10 exons [1–3], was first identified as the causative gene for GA [4] [5]. The *OAT* gene plays a critical role in ornithine metabolism by catalyzing the conversion of ornithine and *α*‐ketoglutarate into glutamate and glutamate‐*γ*‐semialdehyde [6]. Biallelic pathogenic variants in *OAT* result in decreased or absent enzyme activity, leading to elevated concentrations of ornithine in plasma and urine. This hyperornithinemia is toxic to the retinal pigment epithelium (RPE), ultimately causing progressive degeneration and atrophy of the choroid and retina. Clinically, the most common symptoms include night blindness and peripheral visual field constriction due to progressive chorioretinal degeneration [7]. Visual acuity often worsens with macular involvement. Several treatment approaches have been attempted in patients with GA to lower serum ornithine levels and potentially slow chorioretinal atrophy progression. These include an arginine‐restricted diet [8] [9], low‐protein diet [10], and vitamin B6 supplementation[11] [12]. However, due to the ultrarare nature of the disease, most ophthalmologists may never encounter a case in their careers.

In this study, we report a patient with a homozygous *OAT* variant who was diagnosed with GA in her 20s and was unable to continue treatment due to loss to follow‐up.

## 2. Case Presentation

A 21‐year‐old female patient (JU1902) from South Asia was referred to The Jikei University Hospital from a previous hospital for clinical and genetic evaluation due to decreased visual acuity, suspected to be caused by inherited retinal dystrophy. She had no significant past medical history. Information regarding parental consanguinity and sibling composition was unavailable. At the first ophthalmic examination, her best‐corrected visual acuity (BCVA) was 0.4 in the right eye (OD) and 0.5 in the left eye (OS). Refraction was −2.75 diopters in OD and −3.25 diopters in OS. Slit‐lamp examinations showed no anterior segment abnormalities, although posterior subcapsular cataracts were observed.

**Figure 1:**
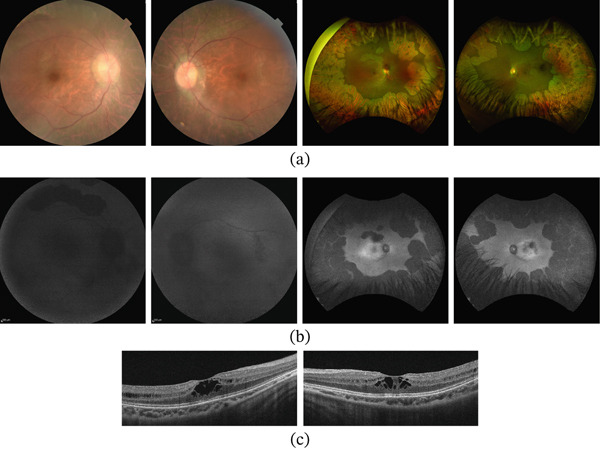
Multimodal retinal imaging findings. (a) Fundus photography reveals confluent chorioretinal atrophy in the peripheral retina as well as around the arcade vessels (superiorly in the right eye). (b) Fundus autofluorescence imaging demonstrates hypoautofluorescence corresponding to the areas of chorioretinal atrophy. (c) Optical coherence tomography reveals multiple intraretinal cystic spaces with preservation of the outer retinal layers, including the ellipsoid zone.

**Figure 2:**
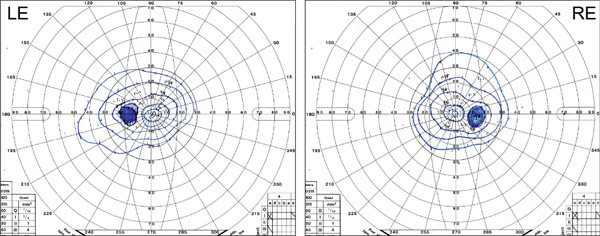
Goldmann perimetry findings. Goldmann perimetry demonstrates markedly constricted visual fields, with the I‐2 isopter restricted to less than 5° and the I‐4 isopter within 10°–20°.

Fundus examination using an ultrawidefield imaging system (Optos, California; Optos, Dunfermline, Scotland, United Kingdom) revealed confluent chorioretinal atrophy in the peripheral retina as well as around the arcade vessels (superiorly in OD) (Figure 1A). Fundus autofluorescence imaging (FAF; Spectralis HRA; Heidelberg Engineering, and Optos California) demonstrated hypoautofluorescence corresponding to the areas of chorioretinal atrophy (Figure 1B). Optical coherence tomography (OCT; Cirrus HD‐OCT 5000, Carl Zeiss Meditec AG, Dublin, California, United States) showed multiple intraretinal cystic spaces, with preservation of the outer retinal layers, including ellipsoid zone (Figure 1C). Goldmann perimetry (GP; Haag‐Streit, Bern, Switzerland) revealed markedly constricted visual field, with the I‐2 isopter restricted to less than 5° and the I‐4 isopter within 10°–20° (Figure 2). Full‐field electroretinography (FF‐ERG) using a light‐emitting diode built‐in electrode (LE‐4000, Tomey, Nagoya Japan) was conducted in accordance with the standards of the International Society for Clinical Electrophysiology of Vision [13]. The detailed FF‐ERG methodology has been previously described [14] [15]. Compared with age‐matched controls with no retinal disease [16], the patient′s ERG responses were nonrecordable under all conditions: dark‐adapted (DA) 0.01, DA 3.0, DA 10.0, light‐adapted (LA) 3.0, and LA 3.0 flicker.

Biochemical testing revealed markedly elevated serum and urinary ornithine concentrations: 775.8 nmol/mL (reference range: 31.3–104.7) and 3353 nmol/mL (reference range: 0–45.1), respectively. Based on these clinical findings, the patient was diagnosed with GA.

## 3. Molecular Genetic Analysis and Follow‐Up

**Figure 3:**
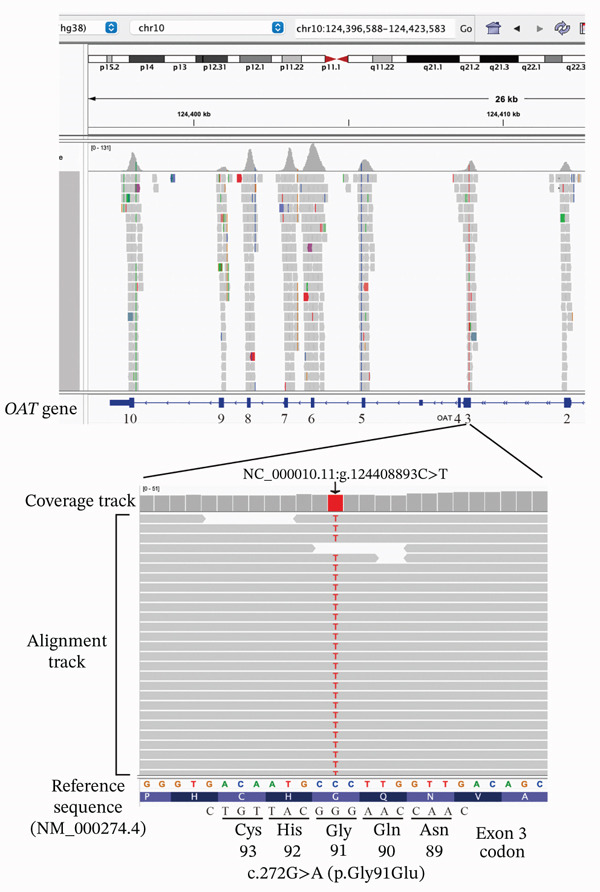
Next‐generation sequencing findings. Visualization using the Integrative Genomics Viewer confirms that the patient (JU1902) is homozygous for a c.272G > A (p.Gly91Glu) variant in exon 3 of the *OAT* gene (NM_000274.4). The corresponding genomic position based on the GRCh38 reference assembly is NC_000010.11: g.124408893C > T.

To determine the underlying cause of the disease, molecular genetic analysis was performed. The study protocol was approved by the institutional Review Board of The Jikei University School of Medicine (Approval Number 24–231 6997) and was conducted in accordance with the tenets of the Declaration of Helsinki. Written informed consent, including publication of clinical data and images, was obtained from both the patient and her father. Peripheral blood samples were collected from the female proband and her father, and genomic DNA was extracted from peripheral leukocytes using the GentraPuregene Blood kit (Qiagen, Hilden, Germany). Whole‐exome sequencing (WES) was conducted by Macrogen Japan (Tokyo, Japan), and the methodology has been previously described in detail [17] [18]. After applying a series of filtering steps, visualization using the Integrative Genomics Viewer revealed that the patient (JU1902) was homozygous for a c.272G > A (p.Gly91Glu) variant in the *OAT* gene (NM_000274.4) (Figure 3). A summary of the identified variant, including its genomic position and predicted effect, is shown in Table 1.

**Table 1:** Summary of the identified *OAT* variant in this study.

Chr	Position (GRCh38)	Allele (REF/ALT)	rs number	Nucleotide change	Gene	Amino acid change	Exon	ToMMo 61KJPN ALT freq	gnomAD v2.1 ALT freq			In silico analysis		ACMG criteria	ACMG criteria	Reference
									Total population	South Asia population	East Asia population	Polyphen‐2	SIFT	Classification	Applicable criteria	
10	124,408,893	C/T	rs386833603	c.272G > A	*OAT*	p.Gly91Glu	3	0.000	0.000	0.000	0.000	Probably damaging (1.000)	Deleterious (0.00)	Likely pathogenic	PS1, PM2, PP3	PMID: 22182799

Abbreviations: ACMG, American College of Medical Genetics and Genomics; ToMMo 61KJPN, Tohoku Medical Megabank Organization 61,000 Japanese individuals.

To confirm the variant, Sanger sequencing was performed using the following primers targeting exon 3 of the *OAT* gene: forward primer 5 ^′^‐TAGGCATTCAGAGGGCTTGC‐3 ^′^ and reverse primer 5 ^′^‐ACTCCAGGGCTCAAAGACTC‐3 ^′^. This confirmed that the patient was homozygous for the c.272G > A (p.Gly91Glu) variant, whereas her father was heterozygous for the same variant.

Following the clinical and genetic diagnosis, vitamin B6 supplementation was initiated at a dose of 100 mg/day for 2 months. However, the serum ornithine level remained markedly elevated at 930.5 nmol/mL (reference range: 31.3–104.7 nmol/mL). Although we considered increasing the vitamin B6 dosage to 200 mg/day and starting an arginine‐restricted diet, the patient voluntarily discontinued follow‐up visits and was subsequently lost to follow‐up.

## 4. Discussion

In this study, we described clinical features of a female patient with GA who carried a homozygous variant (p.Gly91Glu) in the *OAT* gene and was unable to continue treatment.

Common clinical features of GA patients are as follows: (1) night blindness, decreased visual acuity, and constricted visual fields; (2) sharply demarcated circular areas of chorioretinal atrophy in the peripheral retina, macular edema, and markedly reduced rod and cone responses on FF‐ERG; and (3) elevated serum and urinary ornithine levels. These features were consistent with the ophthalmological and systemic findings observed in our patient. To the best of our knowledge, only one other case involving a 10‐year‐old female with the homozygous p.Gly91Glu variant has been reported [19]. That patient exhibited similar findings to our case, including reduced visual acuity, characteristic fundus findings, and multiple intraretinal cystic spaces on OCT [19]. However, she also presented with high myopia, significant astigmatism, and hearing impairment [19].

Although no standardized treatment for GA has been established, reducing serum ornithine concentrations is believed to slow disease progression based on the hypothesis that elevated ornithine levels exert toxic effects on the RPE. Vitamin B6, a coenzyme of OAT, may help lower serum ornithine levels. Additionally, an arginine‐restricted diet may be effective in cases with insufficient response to vitamin B6 alone. In the present case, serum ornithine levels remained elevated despite supplementation with 100 mg/day of vitamin B6. Therefore, we decided to increase the dose to 200 mg/day and concurrently initiate an arginine‐restricted diet to enhance the therapeutic effect.

Follow‐up was discontinued by the patient. The reasons were considered to be as follows: (1) insufficient explanation provided to the patient, likely due to language barriers as English was not her first language and discussions about the therapy were conducted in a nonnative language without the aid of a professional interpreter, (2) a lack of therapeutic education for GA that takes into account cultural factors in South Asia, including religion‐associated dietary practices, and (3) a lack of communication with her parents. GA typically presents during childhood due to visual impairment. However, in this case, the patient was 21 years old at the time of diagnosis and received information regarding the disease and treatment alone. These factors may have contributed to an inadequate understanding of the long‐term visual prognosis and the potential benefits of reducing serum ornithine levels, as well as the importance of regular ophthalmologic and systemic evaluations. This case highlights the need for comprehensive care involving not only the patient but also family members, physicians, nurses, and dietitians.

Currently, there is no clear consensus on the efficacy of treatments such as vitamin B6 supplementation and dietary therapy in halting disease progression. Although some reports have demonstrated slowed progression and improved visual function with these interventions [20–22], others have found no significant benefits [23] [24]. Furthermore, even among patients who initially respond favorably, the long‐term visual prognosis remains uncertain. Based on such findings, some studies have concluded that diet therapy alone may not be effective in altering disease progression [21] [22].

Recent studies using mouse models have shown that intraocular injection of the *OAT* gene via adeno‐associated virus vectors can reduce serum ornithine levels and improve both retinal function and structure [25] [26]. Future therapeutic approaches may aimed at suppressing chorioretinal atrophy progression and restore visual function to some extent. Furthermore, emerging therapies may have the potential to obviate the need for regular blood or urine monitoring and subsequent treatment adjustments based on these results, thereby reducing patient attrition from long‐term care. In the present case with poor response to conventional treatment and feeling anxious about disease progression, such therapies may contribute not only to suppressing disease progression but also to facilitating long‐term follow‐up.

In conclusion, we presented a case of GA that was first diagnosed in adulthood. This case underscores the importance of continued treatment and follow‐up in adult patients with GA, especially those who may lack adequate familial or social support. Forming a patient‐centered multidisciplinary team, including professional interpreters and cultural liaisons, is essential for ensuring patient understanding, treatment adherence, and long‐term visual preservation.

## Author Contributions

Hitomi Taniai: conceptualization, writing—original draft. Kei Mizobuchi: conceptualization, data curation, supervision. Takaaki Hara: conceptualization, data curation, writing—review and editing. Takaaki Hayashi: conceptualization, data curation, writing—review and editing. Tadashi Nakano: conceptualization, writing—review and editing.

## Funding

This study was supported by the Japan Society for the Promotion of Science (JSPS) KAKENHI Grants (JP25K20191 and JP24K12771), and by the Ministry of Health, Labour and Welfare (MHLW) Research on Rare and Intractable Diseases Program Grants (JPMH23FC1043 and JPMH23FC1056).

## Consent

Written informed consent was obtained from the patient and her father for genetic testing and publication of clinical data and images.

## Conflicts of Interest

The authors declare no conflicts of interest.

## Data Availability

The data that support the findings of this study are available from the corresponding author upon reasonable request.
